# Diet Composition and Feeding Strategies of the Stone Marten (*Martes foina*) in a Typical Mediterranean Ecosystem

**DOI:** 10.1100/2012/163920

**Published:** 2012-04-24

**Authors:** Dimitrios E. Bakaloudis, Christos G. Vlachos, Malamati A. Papakosta, Vasileios A. Bontzorlos, Evangelos N. Chatzinikos

**Affiliations:** ^1^Laboratory of Wildlife Ecology and Management, Department of Forestry and Natural Environment Management, Technological Educational Institute of Kavala, 1st km Drama-Mikrohori, 661 00 Drama, Greece; ^2^Department of Wildlife and Freshwater Fisheries, Faculty of Forestry and Natural Environment, Aristotle University of Thessaloniki, P.O. Box 241, 540 06 Thessaloniki, Greece; ^3^Hunting Confederation of Greece, 8 Fokionos Street, 105 63 Athens, Greece; ^4^4th Hunting Federation of Sterea Hellas, 8 Fokionos Street, 105 63 Athens, Greece

## Abstract

Stone martens (*Martes foina*) are documented as generalist throughout their distributional range whose diet composition is affected by food availability. We tested if this occurs and what feeding strategies it follows in a typical Mediterranean ecosystem in Central Greece by analysing contents from 106 stomachs, seasonally collected from three different habitats during 2003–2006. Seasonal variation in diet and feeding strategies was evident and linked to seasonal nutritional requirements, but possibly imposed by strong interference competition and intraguild predation. Fleshy fruits and arthropods predominated in the diet, but also mammals and birds were frequently consumed. An overall low dietary niche breadth (*B*_A_ = 0.128) indicated a fruit specialization tendency. A generalised diet occurred in spring with high individual specialisation, whereas more animal-type prey was consumed than fruits. A population specialization towards fruits was indicated during summer and autumn, whereas insects were consumed occasionally by males. In those seasons it switched to more clumped food types such as fruits and insects. In winter it selectively exploited both adult and larvae insects and partially fruits overwinter on plants. The tendency to consume particular prey items seasonally reflected both the population specialist behaviour and the individual flexibility preyed on different food resources.

## 1. Introduction

The stone marten (*Martes foina* Erxleben, 1777) is one of the most widely distributed mustelid in the Eurasian region, ranging west from Central and Southern Europe to the East in Mongolia, Afghanistan, and Tibet [1]. It is a strictly nocturnal animal, but it could be diurnal during summer [2] living on deciduous woodlands, wooded margins [3], and commonly reported to be found in towns and villages [4–7]. Its population is stable across its range [8]; however, the legal persecution remains a possible threat for its number.

The stone marten has been referred to as a generalist, and its diet is well known in many European countries, mainly in the central [9–11] and southwestern parts of its distribution [12–15]. Its wide spectrum of food types exploited allows the species to occur in variable environments, from undisturbed forests to human settlements. In addition, the numerous studies carried out on the feeding habits of the stone marten note its opportunistic feeding behaviour and that it feeds on fruits, small mammals, insects, birds, reptiles, carrion, and domestic garbage [4, 9, 12, 16–19], but its diet composition is likely affected by regional and seasonal food-type availability [1, 15, 19, 20] and abiotic factors [10], as well as by interspecific competition [21]. Furthermore, there are many studies about its biology and feeding habits suggesting the importance of fruit consumption in its diet [2, 14, 21, 22], as well as reporting the significant contribution of this mammal at the potential improvement of forested areas by enhancing the flora composition with seed dispersal [5, 23, 24]. The stone marten has a terrestrial life, but it is documented as an arboreal species searching for prey on shrubs and/or trees. Its preference for both terrestrial and arboreal prey suggests a relationship with morphological adaptations, such as carnivore dentition, small body size, and long and powerful talons [3]. Its ability to climb allows it to use this wide spectrum of food resources, ranging from fruits to arboreal small mammals, birds, and their eggs.

The study of food habits is applied in wildlife species to describe the dietary composition, to compare diets among geographical regions or among seasons, and to assess the nutritional value of the diet [25]. This information is important to determine the ecological dietary breadth or the “niche” of an animal in the ecosystem and to understand its foraging behaviour, habitat use, and population dynamic, as well as being crucial in order to assess its likely impact on species with ecological and/or sport hunting interest [26]. In most food habit studies the numerical percentage (%N) and the percentage of frequency of occurrence (%F) in combination with the percentage volume or weight of prey items were used in analyses of faecal or stomach contents in mammals [26–28]. Recently, a technique based on stomach contents which includes two of the aforementioned parameters (%F and %N) has been used to explore prey importance, feeding strategy, and the inter- and intraindividual components of niche breadth in predatory fishes [29].

Most of the stone marten's diet studies have analyzed faecal using the percentage of frequency of occurrence. Here, we attempted to estimate the seasonal food habits analyzing stomach contents from Central Greece, where no other dietary study had been carried out before. This approach facilitates investigation into dietary differences between sexes. Thus, the main purposes of this study were (a) to investigate if variation over habitats, seasons, and sexes in prey types taken by the stone marten exist and (b) to gain a better understanding of its food niche characteristics both at the individual and at the population level in its southern distributional range. Both of these can help us to ascertain whether the species is a carnivore or its diet turns to frugivory in a typical Mediterranean ecosystem.

## 2. Materials and Methods

### 2.1. Study Area

Our study area, covering 495,000 Ha, is situated in Central Greece (38°44′–38°59′ N, 22°02′–22°37′ E). Elevations range from 180 to 1,826 m, and the climate is characterized by cold, wet winters and hot, dry summers, with mean annual precipitation ranging between 542 and 1,100 mm and mean annual ambient temperatures over most of the study area averaging 6–17°C. Most of the study area is nonforested land. The dominant habitat type is agricultural land (56.17%) which occurs primarily in extended plains on low altitudes. In the field margins, there are many shrub species such as *Rubus* spp., *Prunus* spp., *Pyrus* spp., and so forth. Shrublands and grasslands (28.33%) contain a variety of plants (*Quercus coccifera*, *Juniperus* spp., *Fragaria vesca*, *Brachypodium sylvaticum,* etc.) mainly on low hills with a mid-relief terrain. Oak forest (14.59%) contains various *Quercus* spp. which are common dominants on higher altitudes with high-relief topography. Large population not only of the red fox (*Vulpes vulpes*) but also of game species such as the European hare (*Lepus europaeus*), the wild boar (*Sus scrofa*), and the rock partridge (*Alectoris graeca*) occupy the study area. Most of the study area has heavy livestock grazing by goat (*Capra hircus*) and sheep (*Ovis aries*).

### 2.2. Stomach Analysis

A total of 106 stomachs mainly hunted animals collected between April of 2003 and March of 2006. Stomachs were sorted according to year, season (spring: March–May, summer: June–August, autumn: September–November, and winter: December–February), gender, and habitat type (farmland, shrubland, and oak forest). Fourteen stomachs were found empty, and they were not included in the dietary analysis. The content of each stomach was analyzed under a dissecting scope and sorted into one of the following prey groups: mammals, birds, reptiles, amphibians, arthropods, molluscs, other invertebrates (molluscs and earthworms), plants, and others (i.e., paper, plastic, string, etc.). All prey items in the stomachs were identified to the lowest taxon possible. The identification was conducted by comparing hairs, teeth, feathers, scales, bones, and seeds by reference collection [30, 31]. We used two common techniques to analyze the diet composition, the percentage of frequency of occurrence (%F = number of stomachs containing prey *i*/total number of stomachs × 100) and the percentage of numerical abundance (%N = number of prey *i*/total number of prey items × 100) [1]. Furthermore, we evaluated the feeding strategy and prey importance using the %F of different prey types plotted against the percentage of prey-specific abundance (%P = number of prey *i*/total number of prey items only in stomachs with prey *i* × 100) (Figure 2(f)) (see Amundsen et al. [29] for detailed description).

We calculated dietary breadth of the stone marten using the Levins standardized equation for food niche [32] *B*_A_ = [(1/∑*p*_*i*_^2^) − 1]/(*n* − 1), where *p*_*i*_ = proportion of occurrence of each prey category in marten's diet and *n* = number of prey categories in stone marten's diet. *B*_A_ values range between 0 and +1, indicating narrow food niche (specialist) when value is close to 0 and broad diet niche (generalist) when the value is close to +1. We used numerical data based on pooled prey categories for the seasonal and overall assessment of the stone marten dietary niche breadth.

We calculated dietary overlap (*O*) between the two sexes using Pianka's [33] modification to the MacArthur and Levin measure of niche overlap [32] *O* = ∑  *p*_*ij*_ *p*_*ik*_/√∑*p*_*ij*_^2^∑*p*_*ik*_^2^, where *p*_*ij*_ and *p*_*ik*_ are the proportions prey class *i* comprised of the diets of the *j* (male) and *k* (female) stone marten genders. Niche overlap values range from 0, for no overlap, to +1, for complete overlap.

### 2.3. Statistical Analysis

We used the log-likelihood ratio *G*-test to analyze the frequency of occurrence of each food group according to years, seasons, and habitat types, as this test has more advantages over the chi-square [34]. Because prey groups did not differ among the three study years (*P* > 0.05) and among the three habitat types (*P* > 0.05), values of the frequency of occurrence were pooled for further analyses. We used log-linear analysis to test for overall interactions among six prey groups, four seasons, and two sexes. Frequencies of the occurrence of six prey groups were used in the log-linear analysis, because reptiles and amphibians were pooled together as well as other invertebrates and others. We tested for the interaction effect among terms of categorical variables using the 95% of confidence intervals criterion. When values of parameter estimation did not contain between the lower and upper 95% of confidence interval, we assumed that the contribution of the parameter (*λ*) to the model was significant [35]. In addition, we tested for differences within each food group, when possible, using the chi-square test for contingency tables.

All statistical analyses were performed using the statistical package SPSS (release 15.0 for windows), and statistical tests were significant if *P* < 0.05.

## 3. Results

### 3.1. Diet Composition

In total 14 stomachs were empty, and the proportion of empty stomachs varied significantly between sex and season (2 × 4 contingency table: *χ* ^2^ = 10.611, d.f. = 3, *P* = 0.014). The highest number of empty stomachs was found in spring (50%), and it corresponded positively to that of males (64.3%) than that of females (35.7%).

A total of 1,025 prey items were recognised in the stomachs of the stone marten, including 21 species, 7 genera, 12 families/orders, and unidentified prey items. Nine major prey groups were identified in the stomach contents (Table 1). Arthropods constitute the most frequently consumed food group, which was observed in 60.9% of the stone marten stomachs, followed by fruits (%F = 55.4), mammals (%F = 30.4), birds and birds' eggs (%F = 20.7), reptiles (%F = 13.01), and molluscs (%F = 7.6). Other prey groups, such as amphibians and earthworms, were almost scarcely consumed. Among arthropods, the Orthoptera (mainly species from families of Acrididae, Gryllotalpidae and Tettigoniidae), the Myriapoda, the Coleoptera, and the Lepidoptera were best represented in terms of %F, and, among plants, fruits of mulberries (*Morus alba*), wild pears (*Pyrus amygdaliformis*), vegetable remains, and grapes (*Vitis vinifera*) were observed in most stomachs analyzed. Among mammals, the southern vole (*Microtus levis*) and the white-toothed shrew (*Crocidura suaveolens*) occurred in the diet more frequently than other small mammals, while the European hare was a rare prey. In addition, the domestic sheep and the edible dormouse (*Glis glis*) accounted for a relatively high proportion of the stone marten diet.

### 3.2. Seasonal Variation

**Figure 1:**
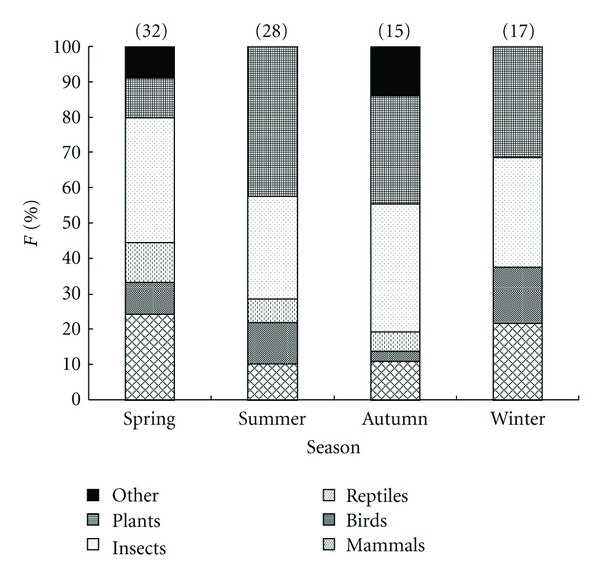
Seasonal percentage of frequency of occurrence (%F) of prey groups in the stone marten diet, in Central Greece during 2003–2006. Numbers above columns are sample sizes.

In terms of seasonal diet composition, there emerged a significant variation using the log-linear analysis (Table 2). First, the log-linear analysis revealed a significant interaction between season and gender (*P* = 0.0115). That is, greater numbers of stomachs of males were collected during summer and winter in comparison to females, there were similar proportions during spring, whereas there were fewer male stomachs in autumn than females'. Second, there was observed a significant seasonal variation in the proportion of food groups in the diet of stone martens (*P* = 0.0003) (Figure 1). The animal groups were especially dominant in the diet during spring (83.3%), they reduced to the lowest proportion during the summer (51.9%), and then increased gradually from 56% in autumn to 63% in winter in terms of frequency of occurrence. Furthermore, the significant contribution of the prey groups was different among the four seasons (Table 3). Insects occurred evenly throughout the year in the stone marten diet. Fruits were observed less frequently than expected during spring (*λ* = −0.990) while they were represented with higher frequencies than expected during summer (*λ* = 0.570). Mammals were present in higher proportions of stomachs during spring (*λ* = 0.518). Birds were found less frequently during autumn (*λ* = −0.851) but in higher frequencies during winter (*λ* = 0.666). Finally, both reptiles and amphibians were not observed in the stone marten diet during winter (*λ* = −1.027). There was also a variation in diet composition within food groups. Within insects, Coleoptera were consumed in similar proportions throughout the year (*χ* ^2^ = 0.254, d.f. = 3, *P* = 0.968), but adult insects were found in the stomachs during spring and summer while larvae were found during autumn and winter. Myriapoda were uniformly found in stomachs throughout the year. Lepidoptera were observed in high proportions during spring (*χ* ^2^ = 22.745, d.f. = 3, *P* < 0.001), whereas Orthoptera were consumed in high proportions during summer. Similarly, there was observed a seasonal fluctuation within fruits. Mulberries were the most consumed food in summer, grapes in autumn and early winter (*χ* ^2^ = 84.09, d.f. = 3, *P* < 0.001), plums (*Prunus spinosa*) in winter, while vegetable remains and wild pears were found in the stomachs in similar proportions throughout the year (*P* > 0.05). Finally, according to log-linear analysis of diet composition, it was found to be relatively homogeneous between the two sexes (*P* = 0.57).

Dietary niche breadth of the stone marten pooled across the study years was relatively low (*B*_A_ = 0.128). It was higher in spring (*B*_A_ = 0.317), then decreased gradually in summer (*B*_A_ = 0.101) and in autumn (*B*_A_ = 0.058), and increased in winter (*B*_A_ = 0.303). Similar pattern was observed both for male and female dietary niche breadth. Females had higher values than males in spring (0.450 versus 0.336), in autumn (0.103 versus 0.056), and in winter (0.520 versus 0.299), whilst males had slightly higher values than females only in summer (0.109 versus 0.086). However, the overall dietary niche breadth was higher in male (*B*_A_ = 0.156) than in female (*B*_A_ = 0.107).

Dietary niche overlap between the two genders was extremely high (*O* = 0.986). However, their food niche overlap was relatively low in spring (*O* = 0.856).

### 3.3. Feeding Strategy

**Figure 2:**
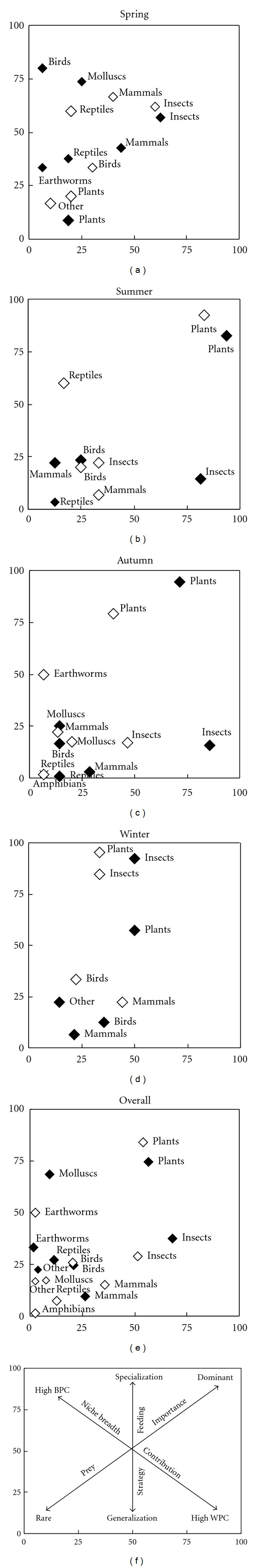
Seasonal (a)–(d) and overall (e) feeding strategy of the male (filled symbols) and the female (open symbols) stone martens in Central Greece, (f) graph redrawn from Amundsen et al. [29]. *X*-axis represents %F (frequency of occurrence), and *Y*-axis %P (prey-specific abundance).

Both male and female stone martens exhibited a similar pattern in their feeding strategies (Figure 2(e)). Furthermore, both sexes showed an overall specialisation on fruits. At the individual level, there was observed a tendency towards a specialised feeding strategy for both sexes, as some prey points were located in the upper half of the diagrams, and this pattern was found for all seasons (Figures 2(a)–2(d)). At the population level, two discernible feeding strategies were observed for both sexes, firstly, a relative population generalisation in spring (Figure 2(a)) and, secondly, a population specialisation in summer (Figure 2(b)), autumn (Figure 2(c)), and winter (Figure 2(d)). The stone marten was relatively generalised as a whole population in spring, as all prey points were located below the diagonal from the lower right to the upper left corner. However, some prey types (e.g., birds and molluscs) were consumed by a few males displaying specialization (high interphenotype component). These types of prey consumed had a high prey-specific abundance value, but they appeared in low frequency of occurrence in the diet, resulting in a relatively narrow niche breadth only in a limited fraction of the population during the spring. 

The population specialisations of both sexes were demonstrated by the prey points being positioned on the upper right part of the graph. In summer and autumn, the population specialisation of both sexes was directed to fruits, whereas in winter fruits and insects were the dominant prey taxa of the population specialisation for both male and female stone martens. The specialisation in those three seasons was almost more pronounced for male than for female stone martens. However, there was observed a high intraphenotype contribution to the male's niche breadths during summer and autumn, as insects appeared in the lower right part of the diagrams. Insects were consumed occasionally by most males during summer and autumn, reflecting a relatively wide niche breadth for this gender.

## 4. Discussion

### 4.1. Diet Composition and Feeding Strategies

In our study the stone marten is a polyphagous mesopredator that consumes a wide spectrum of food types ranging from fruits to invertebrates and small vertebrates and occasionally carrion, as are most of the species of the genus Martes [3, 18, 36]. However, our results suggest that the stone marten in our study area principally fed on insects (%F = 60.9) and to a lesser degree on fruits (%F = 55.4), and this pattern was consistent both interannually (among the three study years) and spatially (among the three main habitat types studied). Small mammals, birds, and birds' eggs constituted a significant part of the diet, whereas reptiles, amphibians, molluscs, and earthworms were of minor importance and thereby may be considered as occasional food. Although insects represent the most important food type in our area, with a Mediterranean character in climate and a heterogeneous landscape, only few studies in the Mediterranean basin have shown similar findings (Portugal: [37], Spain: [16], Italy: [13]), but others have shown that this prey group was not always the case (Portugal: [38], Spain: [12, 21, 39], France: [10, 22], Italy: [19, 20]). Most of the latter authors found the mammalian prey group to be the dominant one in the stone martens' diet, similar to what has been reported in other dietary studies in some Central and Northern European countries (Romania: [9], Czech Republic: [40], Germany: [17], Luxemburg: [41]). In our study area, which is characterised by a mosaic of natural habitats with oak forests, shrublands, and grasslands, insects are favoured both in numbers and diversity due to lack of agrochemicals. Furthermore, insects and especially grasshoppers were found in high densities in agricultural farmlands mainly in non-intensively cultivated crops, possibly due to the low use of insecticides (personal observations). In addition, it is evident that, as the stone marten can utilise a wide variety of habitats [42], it is not surprisingly that insects, which occurred in high numbers in most of natural and seminatural habitats in the study area, had both a consistent (Table 3) and a high presence in its diet (Table 1).

The second most important food group was fruits mainly from wild trees and shrubs, but also cultivated fruits were included in the stone marten's diet. Fruits have been reported as the main food type of the marten's diet in most studies carried out in Central and Northern European countries (see Clevenger [18]), as well as in a few cases in the Mediterranean [2, 21]. In our study area, wild fruits are available from early summer (e.g., mulberries) until late winter. Trees and shrubs which produce fruits are common species in the understory of broadleaved forest, but they especially occur along rain water gullies, while shrubs comprise the main component in shrubland and grassland habitat types in the Mediterranean region. Furthermore, the human-altered agricultural environment studied here was dominated by wild shrubs (e.g., *Rubus* spp., *Prunus* spp., *Ficus* spp., etc.) and trees (*Pyrus* spp., *Morus* spp.) along the field margins, whereas extended agricultural areas on hillsides are covered by vineyards. Thus, fruits are almost always available in high numbers across the study area and thereby may constitute the food for a wide range of animals including the stone marten [12, 15, 43].

Our results demonstrate that a seasonal variation in the stone marten's diet apparently exists. This pattern could be related to the species' nutritional requirements throughout the year, although inter- and intraspecific competition could be involved [44]. In spring, the stone marten consumed a high disproportionate percentage (up to 84%F) of small-sized animals to fulfil its highly energy requirements that season, including insects, mammals, birds, reptiles, while in contrast, fruits and vegetable remains were not encountered frequently in its diet. In particular, small mammals seemed to constitute an important component in the diet of stone martens, as this was revealed by the log-linear analysis (Table 3 and Figure 1). Most of small mammals consumed were shrews, voles, and wood mice, but also carrion from dead domesticated animals was taken. During that season adults and juveniles rodents are encountered at a high rate and are easy to capture [3]. In addition, arthropods, included Myriapoda, Lepidoptera, and Coleoptera, composed a high proportion of the marten's diet [10]. Fruits made up a negligible portion of the diet in spring, and most of those found in stomachs were probably collected from human refuse. In spring the stone marten exhibited a generalist feeding strategy as was expected, consuming a wide range of prey types. In our study this was suggested by both the broad diet niche breadth index (*B*_A_ = 0.317) and the graphical representation of prey points (Figure 2(a)). However, in spring a high interindividual phenotype specialization emerged [45], as birds, reptiles, and molluscs have been eaten by relatively few individuals. It has been suggested that specialization of a generalist species could be attributed to interspecific competition as the result of a facultative behavioural change in certain resource use [46]. Furthermore, asymmetric intraguild predation among mammalian carnivores can have effects analogous to those of competition [46–48]. In our study, the stone marten population suffered a high predation rate from the red fox year-round, but especially in spring [49]. Similar findings were reported in other studies where the stone marten was found to be a prey of other mesopredators, like the red fox [39]. Another explanation for the high interphenotype component could be the interference competition (intrapopulation competition) that limits the range of food resources utilised by territorial individuals within their breeding space [50, 51]. Indeed, this may occur in our stone marten population due to the highly heterogeneous landscape in the study area [52], and; thus, different individuals are specializing on those food resources that are abundant within their home ranges [53], reflecting a variation in behavioural or physiological traits of individuals that determine resource-use efficiencies and different preferences [45, 54]. Furthermore, it has been suggested that strong interference competition leads to decreased rates of resource (food) intake per individual [51], and probably in our study this was the reason why more male stone martens were found with empty stomachs during spring, the critical breeding season [50].

In summer and autumn, fruits and insects became the most important foods in the stone marten diet [55]. In addition, reptiles, amphibians, and birds were consumed during these seasons but to a lesser degree. Both fruits and insects are abundant in the study area during summer. Due to seasonal ripeness of fruits in our study area, the stone marten shifts its diet seasonally to the most abundant species. Early summer mulberries composed the principal food items, whereas during the summer other abundant fruits (e.g., *Ficus* spp., *Pyrus* spp., *Rubus* spp., *Prunus* spp.) were taken, with grapes being dominant during autumn. Insects were also an important prey type taken [1], but Orthoptera predominate over other arthropods in the stone marten's diet during that period [10]. Crickets and grasshoppers (Orthoptera) are very abundant in the central part of Greece, and usually they appear to experience population explosions especially during the summer. In particular, mammals and partly birds did not contribute to the stone marten diet during this period. Although the food resource diversity increases in Mediterranean ecosystems during summer and autumn [52], the dietary niche breadth in our studied stone marten population decreased in both seasons. In addition, both sexes of the stone marten exhibited a specialized feeding strategy, consuming mainly fruits during summer and autumn [1, 18] (Figures 2(b) and 2(c)). However, an increased intraphenotype component to the niche breadth of males demonstrated a generalised diet on insects at the individual level [29]. Although there are no data on the availability of small mammals (rodents) and birds in our study area, we assumed that these animal groups were abundant during summer as they are in environments similar to those in our area [10, 38] and easy to capture by a predator as juveniles and nestlings appeared in high numbers during that season [19, 20, 56]. These prey groups could be considered optimal prey types for stone marten in energetic terms during summer, as the species has to breed due to delayed implantation and; thus, it has to fulfil its high energy demands by the more profitable prey [3, 57]. Surprisingly, we found a high proportion of fruits and insects in the stone marten's diet during summer. Fleshy fruits could be considered suboptimal food for stone marten from a nutritional point of view [44, 51], although they are nutritious and digestible [3]. Similarly, insects could be rated as suboptimal prey as they provide less energy and require a great deal of time for searching and capturing [51]. However, according to the optimal foraging theory, specialization on a less profitable food type can be optimal if the food type is sufficiently clumped [46]. The dietary switching to less profitable food types, such as fruits and insects [21, 37], could arise again by the intense interspecific competition between the red fox and the stone marten in our study area [58]. Therefore, two different scenarios could be associated with specialising the diet of the stone marten during summer. Strong interspecific competition may, on one hand, switch to suboptimal prey types resulting in decreased dietary niche breadth [58]. Alternatively, stone marten displays a specialised feeding strategy at the population level as it expends a great deal of time and energy selectively searching for suboptimal food types [44, 51].

In winter arthropods and fruits were the dominant food types in the stone marten's diet, but also birds were taken in higher proportions than expected (Table 3 and Figure 1). In contrast with other studies where mammals dominated in diet during winter [2, 10, 13, 19, 38], in our studied stone marten population it showed an apparent selectivity for arthropods and fruits [55]. Other studies have also shown that the stone marten fed on birds in winter [2, 20], as did other mustelids [59]. Although insects were not abundant during that season, the stone marten exploited high numbers of this prey items both as adult and larvae beetles (Coleoptera). Furthermore, even when the period of ripe fruit had passed, stone marten consumed high numbers of fleshy fruits overwinter on the plant and those which had remained intact until late season, such as plums and wild pears [40]. In winter, both sexes displayed a relative specialisation for fruits and arthropods (Figure 2(d)), whereas neither intra nor inter-phenotype component at the niche breadth was detected during that season. Even in that season, the specialisation is not pronounced (*B*_A_ = 0.303) as in summer and in autumn; the stone marten could be considered a relative specialist, due to the high contribution of insects and fruits and to the occasional participation of small mammals, birds, and other food items in its diet (Figure 2(d)). Although a broader diet during unproductive environments, as winter, was revealed [51], the stone marten seemed to spend more time and energy searching for insects' larvae digging from fallen woods, demonstrating specialist behaviour during that season.

Finally, in our study the impact of stone marten on economically important wildlife species (i.e., the European hare) or domestic animals could be regarded negligible. In stone marten stomachs, there were occasionally found items of food of unexpected size, such as domestic sheep and goat, and these were nearly taken as carrion.

### 4.2. Specialist or Generalist Mustelid?

A specialist is an animal which exploits efficiently a narrow prey spectrum regardless of its availability [36, 44]. On the other hand, a generalist is an animal which can exploit several alternative prey types according to their availability. In our study, both classic niche breadth indices (*B*_Am_ = 0.156, *B*_Af_ = 0.107) and graphical representation of prey-specific abundance against the frequency of occurrence of prey types suggest that stone marten exhibited a specialised feeding strategy. Furthermore, at the individual and at the population level, it showed a mixed feeding strategy according to seasons. During three out of the four seasons, stone marten indicated a pronounced population specialization while a clear generalization of male individuals was observed during summer and autumn. An evident generalised diet was revealed only during spring, but again a specialised tendency for few food groups was observed in the diet of some individuals. Unfortunately, only one study of the stone marten food habits has shown individual specialization within a generalist population [1]. However, individual specialization has been detected in other species of the family Mustelidae (pine marten *Martes martes*: [60], American marten *Martes americana*: [53], genet *Genetta genetta*: [61], badger *Meles meles*: [62]). Most of the studies conducted on food habits of stone marten have used analysis of faeces, and probably they failed to detect dietary specialization neither at the individual nor at the population level. With the results of our study, we suppose that stone marten exploited heavily one or two food groups year-round [18], and; thus, its tendency towards specialization than generalization is more evident [36].

## 5. Conclusion

In conclusion, this work is the first attempt to investigate both the diet composition and the feeding strategy of stone marten by analysing its stomach contents from mainland Greece. Stone marten shows seasonal differences in diet as well as mixed feeding strategies at least at the local level. The tendency of consumption of particular prey items seasonally, which is not always associated with an increased abundance in the environment, reflects, on one hand, population specialist behaviour, while on the other hand, it shows the individual flexibility on different food resources (intra and interindividual specialization) of this medium-sized mustelid. Possible mechanisms which have driven the stone marten to a more specialised diet both at the individual and at the population level were interference competition and intraguild predation. However, the extent to which stone marten behaves as a specialist, at least locally, under a strong inter- and/or intraspecific competition in mainland Greece could be better clarified by comparing the food habits from similar areas where no intraguild predation occurs.

## Figures and Tables

**Table 1:** Diet composition of the stone marten in Central Greece, during 2003–2006.

Group	Order/family	Species/item	%F	%N
Mammals	Lagomorpha	*Lepus europaeus*	1.09	0.10
	Rodentia	*Apodemus sylvaticus*	1.09	0.10
		*Apodemus mystacinus*	2.17	0.20
		*Glis glis*	3.26	0.29
		*Micromys minutus*	1.09	0.20
		*Clethrionomys glareolus*	1.09	0.10
		*Rattus rattus*	1.09	0.10
		*Microtus levis*	4.35	0.49
	Soricomorpha	*Crocidura leucodon*	2.17	0.20
		*Crocidura suaveolens*	4.35	0.39
	Artiodactyla	*Ovis aries*	4.35	0.39
		*Capra hircus*	1.09	0.10
		Nonidentified mammals	4.35	0.39
		Total mammals	**30.4**	**3.0**
Birds		Nonidentified birds	13.04	1.66
		Eggs	7.61	0.59
		Total birds	**20.7**	**2.2**
Reptiles	Sauria	*Lacerta viridis*	2.17	0.29
		*Podarcis muralis*	1.09	0.10
		Nonidentified lizards	7.61	0.68
	Ophidia	Nonidentified snakes	1.09	0.10
Amphibians		*Rana *spp.	1.09	0.10
		Total herptiles	**14.1**	**1.3**
Arthropods	Coleoptera		13.04	1.56
	Hymenoptera		3.26	0.29
	Lepidoptera		11.96	4.29
	Orthoptera		30.43	3.51
	Myriapoda		15.22	2.34
	Trichoptera		1.09	0.20
	Libellulidae		1.09	0.10
	Arachnida		2.17	0.20
		Nonidentified insects	15.22	11.51
		Total insects	**60.9**	**24.0**
Molluscs		*Helix *spp.	1.09	0.10
		*Arion *spp.	7.61	2.83
Earthworms	Lumbricidae		2.17	0.39
		Total other invertebrates	**9.8**	**3.3**
Plants		*Morus alba*	13.04	17.27
		*Pyrus amygdaliformis*	10.87	1.56
		*Prunus spinosa*	3.26	4.20
		*Prunus *spp.	3.26	0.39
		*Rubus *spp.	1.09	0.10
		*Rosa canina*	2.17	0.20
		*Ficus *spp.	3.26	0.39
		*Amygdalus communis*	1.09	0.10
		*Actinidia polygama*	1.09	0.10
		*Vitis vinifera*	8.70	40.00
		Vegetable remains	9.78	0.98
		*Hordeum* spp.	1.09	0.10
		Nonidentified plants	6.52	0.49
		Total plants	**55.4**	**65.6**
Other		other items	**3.3**	**0.3**

**Table 2:** Log-linear model for frequency of occurrence of prey items in the stone marten diet in Central Greece, during 2003–2006.

Source of variation	d.f.	**χ** ^2^	*P* value
sex × season × food	15	15.74	0.3994
sex × season	3	11.04	0.0115
sex × food	5	3.83	0.5744
season × food	15	41.43	0.0003
sex	1	7.92	0.0049
season	3	8.69	0.0336
food	5	109.03	<0.001

**Table 3:** Parameters (*λ*) of the interaction term season × food in the log-linear model. Bold number indicates significant contribution of the parameter to the model by using the 95% confidence interval criterion.

Prey group	Spring	Summer	Autumn	Winter
Mammals	**0.518**	−0.082	−0.333	−0.103
Birds	−0.187	0.372	−**0.851**	**0.666**
Herptiles^a^	0.545	0.219	0.263	−**1.027**
Arthropods	−0.067	0.008	0.253	−0.194
Plants	−**0.990**	**0.570**	0.262	0.158
Other^b^	0.181	−1.087	0.406	0.500

^a^Reptiles and amphibians.

^b^Molluscs, earthworms, and other food items.
